# Pregnancy in a unicornuate uterus: a case report

**DOI:** 10.1186/1752-1947-8-130

**Published:** 2014-04-29

**Authors:** Donatella Caserta, Maddalena Mallozzi, Cristina Meldolesi, Paola Bianchi, Massimo Moscarini

**Affiliations:** 1 Department of Gynecological-Obstetric and Urological Sciences, University of Rome “Sapienza”, Sant’Andrea Hospital, Via di Grottarossa 1035-1039, Rome 00189, Italy; 2 Department of Gynecological-Obstetric, S. Pietro Fatebenefratelli Hospital, Via Cassia 600, Rome 00189, Italy

**Keywords:** Congenital Müllerian malformations, Congenital uterine anomalies, Pregnancy outcomes, Pregnancy unicornuate uterus

## Abstract

**Introduction:**

A unicornuate uterus accounts for 2.4 to 13% of all Müllerian anomalies. A unicornuate uterus with a non-communicating rudimentary horn may be associated with gynecological and obstetric complications such as infertility, endometriosis, hematometra, urinary tract anomalies, abortions, and preterm deliveries. It has a poor reproductive outcome and pregnancy management is still unclear.

**Case presentation:**

We report a case of a 26-year-old Caucasian woman presenting with a unicornuate uterus with a non-communicating rudimentary horn. The diagnosis of the anomaly was based on two-dimensional and three-dimensional sonography. The excision of her symptomatic rudimentary horn and her ipsilateral fallopian tube was performed laparoscopically. The growth of the fetus was normal. At 20 weeks’ pregnancy, her cervix started shortening and a tocolytic therapy was started. A cesarean delivery was successfully performed at 39 weeks and 4 days’ gestation.

**Conclusions:**

Although the reproductive outcome of women with unicornuate uterus is poor, a successful pregnancy is possible. Routine excision of the rudimentary horn should be undertaken during non-pregnant state laparoscopically, and it would be necessary to screen such pregnancies for the development of intrauterine growth retardation with serial ultrasound assessments of the estimated fetal weight and the cervix length.

## Introduction

Congenital uterine anomalies result from an abnormal formation, fusion or reabsorption of Müllerian ducts during fetal life*.* These anomalies are present in 1 to 10% of the unselected population, 2 to 8% of infertile women and 5 to 30% of women with a history of miscarriages [1]*.* The true population prevalence of congenital uterine anomalies is difficult to assess partly because there are no universally standardized classification systems and partly because the best diagnostic techniques are invasive, therefore, they are rarely applied to low-risk study populations.

The presence of a maternal uterine anomaly is associated with an increased risk of preterm birth, preterm premature rupture of membranes, breech presentation, cesarean section, placenta previa, placental abruption and intrauterine growth retardation (IUGR) [2]. A unicornuate uterus is present in 0.1% of the unselected population. The reproductive performance of women with unicornuate uterus is poor, with a live birth rate of only 29.2%, prematurity rate of 44%, and an ectopic pregnancy rate of 4% [3]. Moreover, women with this anomaly, present rates of 24.3% first trimester abortion, 9.7% second trimester abortion and 10.5% intrauterine fetal demise [4]*.* It has been suggested that first trimester abortion, intrauterine growth restriction, and stillbirths, may be explained by an abnormal uterine blood flow (absent or abnormal uterine or ovarian artery). Second trimester abortions and preterm deliveries are thought to be due to decreased muscle mass in the unicornuate uterus as well as cervical incompetence.

A unicornuate uterus is a type II classification with unilateral hypoplasia or agenesis that can be further subclassified into communicating, no cavity and no horn [5]. A rudimentary horn with unicornuate uterus results from failure of complete development of one of the Müllerian ducts associated with the incomplete fusion of the contralateral one. In 83% of cases, the rudimentary horn is non-communicating and often associated with ectopic pregnancies [6]. Pregnancy in non-communicating rudimentary horn is possible by transperineal migration of sperm or fertilized ovum. It occurs in approximately 1 out of 76,000 pregnancies. The risk of uterine rupture is 50 to 90%, with most ruptures (approximately 80%) occurring by the end of the second trimester [7].

We present a case report of a successful pregnancy in a unicornuate uterus in a Caucasian woman.

## Case presentation

A 24-year-old Caucasian woman came to our observation with a history of dysmenorrhea from the menarche. An ultrasound investigation, including an initial two-dimensional (2D) ultrasound assessment of her pelvis with the selection of the region of interest and the acquisition of a three-dimensional (3D) ultrasound, was performed. The investigation revealed a right unicornuate uterus of the dimensions of 71mm × 33mm × 30mm with an endometrial thickness according to her menstrual phase. At the left side, lining her left ovary, a non-communicating rudimentary horn with an endometrial thickness of 7mm was described, which also accorded with her menstrual phase (Class II B by the American Fertility Society 1988).

Both her ovaries were normal for morphology and volume.

**Figure 1:**
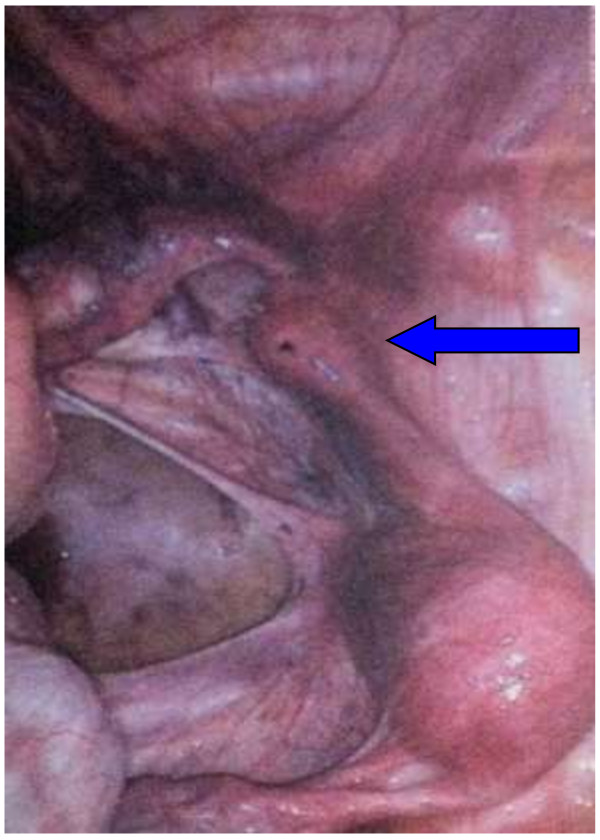
Laparoscopy image of the unicornuate uterus with non-communicating horn (indicated by the arrow).

Because the presence of a cavity in the rudimentary horn is the most important factor leading to complications such as ectopic pregnancy, the treatment of rudimentary horn laparoscopic removal was indicated [8,9] (Figure 1).

The patient became pregnant 18 months later; therefore, blood tests and ultrasound examinations were regularly performed as for a pregnancy in a normal uterus.

Obstetric ultrasound examinations at the first, second and third trimester of her pregnancy showed a normal insertion of the placenta, normal amniotic fluid index and breech presentation.

At 20 weeks’ pregnancy, she came to our observation complaining of lower abdominal pain; an obstetric visit and a cervix ultrasound measurement were performed revealing a cervix length of 34mm.

A tocolytic therapy was prescribed and serial ultrasound measurements of her cervical length were performed.

The tocolytic therapy consisted of 5mg of ritodrine twice a day, with the aim of relaxing the smooth muscle fibers stimulating the beta receptors on the cell membrane.

At 33 weeks’ pregnancy, an intrauterine growth under the normal threshold was detected. A serial growth ultrasound examination was performed confirming a low baby weight (10° percentile) until the last weight estimation at 37 weeks and 4 days’ gestation.

At 39 weeks’ pregnancy, the patient came to our observation complaining of contractions and light vaginal bleeding so an obstetric visit, cardiotocography and ultrasound measurement of her cervix were immediately performed. Both her cervix and vagina were healthy on a pelvic examination; cardiotocography revealed only sporadic uterine contractions and her cervix length was 28mm.

She was invited to return to our obstetric unit so as to perform the cesarean cut at 39 weeks and 4 days’ gestational.

She underwent a cesarean section and she had a healthy baby boy, with an Apgar score of 9 to 10, weight of 3160g and height of 49cm.

The mother had no postsurgery complications.

## Conclusions

Patients who have a unicornuate uterus with rudimentary horn have an increased incidence of gynecologic problems and tend to present, at menarche or later in their life, symptoms such as dysmenorrhea and chronic pelvic pain [10].

This is why patients affected by these symptoms should always be screened with 2D and 3D ultrasound examinations.

Nevertheless ultrasound diagnosis can be missed, particularly in inexperienced hands.

Even though Nanda *et al.*[11] described a successful twin pregnancy in a unicornuate uterus with one fetus in the non-communicating rudimentary horn, many other cases of ruptured non-communicating rudimentary horn pregnancies have been described [12,13].

A unicornuate uterus with rudimentary horn is often associated with ectopic pregnancies and with rupture of the rudimentary horn and, although it is unclear whether or not to remove the rudimentary horn before conception or early in pregnancy, its resection decidedly improves obstetrical outcomes.

Even when a resection of the rudimentary horn is performed, patients with a unicornuate uterus present a higher risk of obstetrical complications, such as first trimester abortion, second trimester abortion, intrauterine growth restriction, preterm delivery and intrauterine fetal demise, and only a few obstetrical risks can be reduced by a particular pregnancy follow up and specific interventions.

According to the current guidelines of the American Congress of Obstetricians and Gynecologists (ACOG) for the management of IUGR [14]*,* it is reasonable to consider serial growth ultrasound examinations in pregnancies at risk of IUGR as in the case of a unicornuate uterus pregnancy.

It is important to consider that sonographic weight estimation appears to be less accurate for fetuses in breech presentation [14] because all uterine anomalies increase the chance of fetal malpresentation.

As for the risk of preterm labor, there are no consistent data that any intervention can delay delivery in women for longer than 24 to 48 hours once they present a preterm labor. For this reason, much attention has been focused on preventive strategies rather than on intervention strategies.

Although several strategies have been proposed, the prevention of preterm birth has been largely unsuccessful [15].

The utility of ultrasound cervix length measurement for assessing the risk of preterm birth has been well documented, with an accepted cutoff value for cervix length of ≤25mm before the 24th week of gestational age.

The predictive value of a negative test is high (92%); this implies that pregnant women who do not have a shortened cervix can be reassured, and unnecessary therapeutic measures can be avoided.

By contrast, cervical cerclage is the best treatment for women with a short cervix (<25mm), and particularly for women with a history of prior midtrimester pregnancy losses due to cervical insufficiency, Therefore, in our case report, a cervical cerclage was considered unnecessary.

Whether progesterone acts by attenuating further cervical shortening is not clear yet.

Accumulating evidence suggests that the myometrial activity associated with preterm labor results primarily from a release of the inhibitory effects of pregnancy on the myometrium rather than an active process mediated through the release of uterine stimulants, and progesterone appears to play a central role.

Recent data suggest that progesterone may be important in maintaining uterine quiescence in the latter half of pregnancy by limiting the production of stimulatory prostaglandins and inhibiting the expression of contraction-associated protein genes (ion channels, oxytocin and prostaglandin receptors, and gap junctions) within the myometrium.

The role of progesterone in later pregnancy, however, is less clear.

In fact, ACOG recommend progesterone supplementation only for prior spontaneous preterm birth and cervical shortening (≤15mm prior to 24 weeks) so we decided not to administer this treatment in our present experience.

Although a premature birth can also be due to premature contractions, a tocolytic therapy is suggested in this situation.

In our case report it was considered useful to perform serial growth ultrasound examinations for assessing a possible IUGR and an ultrasound cervix length measurement to assess the risk of preterm birth, and to prescribe a ritodrine tocolytic therapy when contractions led to shortening of the cervix length.

Our case report shows that by adopting these strategies the prognosis of pregnancy in a unicornuate uterus is not always impaired, although breech presentation, cesarean delivery and prematurity threatens to occur.

Nevertheless, the optimal management approach cannot be clearly stated. Further large observational and prospective studies are essential to investigate the treatments needed during pregnancies in this uterine anomaly.

## Consent

Written informed consent was obtained from the patient for publication of this case report and any accompanying images. A copy of the written consent is available for review by the Editor-in-Chief of this journal.

## Abbreviations

2D: two-dimensional; 3D: three-dimensional; ACOG: American Congress of Obstetricians and Gynecologists; IUGR: Intrauterine growth retardation.

## Competing interests

The authors declare that they have no competing interests.

## Authors’ contributions

DC was responsible for the concept and was the main author who designed the study, MMa analyzed and interpreted the patient’s data and meticulously analyzed the literature, PB followed the patient’s clinical progress, CM performed the surgery and the manuscript was critically reviewed and edited by MMo. All authors read and approved the final manuscript.

## References

[B1] Chan YY Jayaprakasan K Tan A Thornton JG Coomarasamy A Raine-Fenning NJ Reproductive outcomes in women with congenital uterine anomalies: a systematic review Ultrasound Obstet Gynecol 2011 38 371 382 10.1002/uog.10056 21830244

[B2] Hua M Odibo AO Longman RE Macones GA Roehl KA Cahill AG Congenital uterine anomalies and adverse pregnancy outcomes Am J Obstet Gynecol 2011 205 6 558 e1–e5 21907963 10.1016/j.ajog.2011.07.022

[B3] Akar ME Bayar D Yildiz S Ozel M Yilmaz Z Reproductive outcome of women with unicornuate uterus Aust N Z Obstet Gynaecol 2005 45 2 148 150 10.1111/j.1479-828X.2005.00346.x 15760318

[B4] Reichman D Laufer MR Robinson BK Pregnancy outcomes in unicornuate uteri: a review Fertil Steril 2009 91 5 1886 1894 10.1016/j.fertnstert.2008.02.163 18439594

[B5] Grimbizis GF Campo R Gordts S Brucker S Gergolet M Tanos V Li T-C De Angelis C Di Spiezio Sardo A On behalf of the Scientific Committee of the Congenital Uterine Malformations (CONUTA) common ESHRE/ESGE working group Clinical approach for the classification of congenital uterine malformations Gynecol Surg 2012 9 119 129 10.1007/s10397-011-0724-2 22611348 PMC3338910

[B6] Thakur S Sood A Sharma C Ruptured noncommunicating rudimentary horn pregnancy at 19 weeks with previous cesarean delivery: a case report Case Rep Obstet Gynecol 2012 2012 308476 doi:10.1155/2012/308476 23119197 10.1155/2012/308476 PMC3483705

[B7] Rackow BW Arici A Reproductive performance of women with müllerian anomalies Curr Opin Obstet Gynecol 2007 19 3 229 237 10.1097/GCO.0b013e32814b0649 17495638

[B8] Fedele L Bianchi S Zanconato G Berlanda N Bergamini V Laparoscopic removal of the cavitated noncommunicating rudimentary uterine horn: surgical aspects in 10 cases Fertil Steril 2005 83 432 436 10.1016/j.fertnstert.2004.07.966 15705386

[B9] Theodoridis TD Saravelos H Chatzigeorgiou KN Zepiridis L Grimbizis GF Vavilis D Loufopoulos A Bontis JN Laparoscopic management of unicornuate uterus with non-communicating rudimentary horn (three cases) Reprod Biomed Online 2006 12 1 128 130 10.1016/S1472-6483(10)60992-3 16454949

[B10] Khati NJ Frazier AA Brindle KA The unicornuate uterus and its variants. Clinical presentation, imaging findings, and associated complications J Ultrasound Med 2012 31 319 331 22298877 10.7863/jum.2012.31.2.319

[B11] Nanda S Dahiya K Sharma N Aggarwal D Sighal SR Sangwan N Successful twin pregnancy in a unicornuate uterus with one fetus in the non-communicating rudimentary horn Arch Gynecol Obstet 2009 280 6 993 995 10.1007/s00404-009-1028-x 19301025

[B12] Amritha B Sumangali T Priya B Deepak S Sharadha R A rare case of term viable secondary abdominal pregnancy following rupture of a rudimentary horn: a case report J Med Case Rep 2009 3 38 10.1186/1752-1947-3-38 19178737 PMC2640409

[B13] Kanagal DV Hanumanalu LC Ruptured rudimentary horn pregnancy at 25 weeks with previous vaginal delivery: a case report Case Rep Obstet Gynecol 2012 2012 985076 doi:10.1155/2012/985076 22720180 10.1155/2012/985076 PMC3375039

[B14] American Congress of Obstetrics and Gynecology Committee on Practice Bulletins- Obstetrics ACOG practice bulletin: intrauterine growth restriction Obstet Gynecol 2000 95 Suppl 1 1210636492

[B15] Norwitz ER Phaneuf LE Caughey A Progesterone supplementation and the prevention of preterm birth Rev Obstet Gynecol 2011 4 2 60 72 22102929 PMC3218546

